# A comprehensive systematic review of CSF proteins and peptides that define Alzheimer’s disease

**DOI:** 10.1186/s12014-020-09276-9

**Published:** 2020-06-05

**Authors:** Cristina M. Pedrero-Prieto, Sonia García-Carpintero, Javier Frontiñán-Rubio, Emilio Llanos-González, Cristina Aguilera García, Francisco J. Alcaín, Iris Lindberg, Mario Durán-Prado, Juan R. Peinado, Yoana Rabanal-Ruiz

**Affiliations:** 1 Department of Medical Sciences, Ciudad Real Medical School, Oxidative Stress and Neurodegeneration Group, Regional Center for Biomedical Research, University of Castilla-La Mancha, Ciudad Real, Spain; 2 Department of Anatomy and Neurobiology, University of Maryland School of Medicine, University of Maryland, Baltimore, MD 21201 USA

**Keywords:** Alzheimer´s Disease, Biomarkers, Proteomic, Peptidomics

## Abstract

**Background:**

During the last two decades, over 100 proteomics studies have identified a variety of potential biomarkers in CSF of Alzheimer’s (AD) patients. Although several reviews have proposed specific biomarkers, to date, the statistical relevance of these proteins has not been investigated and no peptidomic analyses have been generated on the basis of specific up- or down- regulation. Herein, we perform an analysis of all unbiased explorative proteomics studies of CSF biomarkers in AD to critically evaluate whether proteins and peptides identified in each study are consistent in distribution; direction change; and significance, which would strengthen their potential use in studies of AD pathology and progression.

**Methods:**

We generated a database containing all CSF proteins whose levels are known to be significantly altered in human AD from 47 independent, validated, proteomics studies. Using this database, which contains 2022 AD and 2562 control human samples, we examined whether each protein is consistently present on the basis of reliable statistical studies; and if so, whether it is over- or under-represented in AD. Additionally, we performed a direct analysis of available mass spectrometric data of these proteins to generate an AD CSF peptide database with 3221 peptides for further analysis.

**Results:**

Of the 162 proteins that were identified in 2 or more studies, we investigated their enrichment or depletion in AD CSF. This allowed us to identify 23 proteins which were increased and 50 proteins which were decreased in AD, some of which have never been revealed as consistent AD biomarkers (i.e. SPRC or MUC18). Regarding the analysis of the tryptic peptide database, we identified 87 peptides corresponding to 13 proteins as the most highly consistently altered peptides in AD. Analysis of tryptic peptide fingerprinting revealed specific peptides encoded by CH3L1, VGF, SCG2, PCSK1N, FBLN3 and APOC2 with the highest probability of detection in AD.

**Conclusions:**

Our study reveals a panel of 27 proteins and 21 peptides highly altered in AD with consistent statistical significance; this panel constitutes a potent tool for the classification and diagnosis of AD.

## Background

Due to its high prevalence within the world population, Alzheimer´s disease (AD) is likely the most studied neurodegenerative disease [1]. A variety of studies have characterized AD pathology from a cognitive point of view [2] and/or using neuroimaging approaches [3]. While these approaches are necessary in order to distinguish the various hallmarks of AD progression, there is still a need to identify reliable biomarkers for the effective diagnosis and prognosis of AD.

Most studies aimed at the detection of AD biomarkers have been carried out on biological fluids such as blood or cerebrospinal fluid (CSF) (reviewed in [4–6]). The use of CSF represents the best approach to identify AD biomarkers (reviewed in [6]) since this fluid contacts the brain interstitial fluid directly and thus more accurately reflects biochemical changes related to central nervous system (CNS) processes. Indeed, studies of AD CSF have consistently demonstrated that amyloid-β (Aβ42), total tau (T-tau), and phosphorylated tau (P-tau) constitute CSF biomarkers relevant to AD diagnosis [6–8]. However, the heterogeneity of AD pathology calls for a deeper review of potential AD CSF biomarkers. Biomarker use may eventually help to predict disease progression, from asymptomatic stages to full-blown AD. Accordingly, over 100 different proteomic studies of potential CSF biomarkers in AD have been conducted during the past 15 years.

A number of reviews have been recently published which have examined these past studies and have proposed specific biomarkers for AD in CSF [9, 10]. However, only 3 proteomic reviews with controlled analyses directly comparing biomarkers in AD and control CSF have been conducted thus far. In 2016, Olsson et al. described which of the common biomarkers in both CSF and blood were the most altered in AD. They reported 5 CSF core biomarkers associated with AD [11]. Subsequently, Bastos et al. compiled data from 18 proteomic studies to obtain 309 proteins expressed differentially in CSF obtained from AD patients *vs.* controls [12]. The most recent CSF proteomic analysis, which appeared while we were finishing our study, included 29 studies, of which 25 specifically investigated differences between AD and controls, and reported that 478 proteins exhibited different levels in AD compared to controls [13]. This analysis includes most of the proteins that have been shown to differ between AD and controls. However, these data do not consider the statistical relevance of hits. Furthermore, none of the indicated reviews analyze all tryptic peptides on the basis of specific up- or down- regulation in AD. Therefore, the accuracy of the correlation of these potential protein biomarkers, and their specific tryptic peptides, with actual AD pathology remains unclear.

In the current review, by using various bioinformatics resources, we have generated a consistent data output which compiles protein changes presented in widely different formats in the various original independent studies. First, we searched the literature for unbiased CSF explorative proteomics studies that investigate AD and compiled data from 47 independent published studies, which considerably increases the number of studies collected so far, thereby expanding to 601 the total number of proteins identified in proteomic studies of CSF from AD patient samples. Additionally, we classified those studies as either descriptive or supported by quantitative data in order to exclusively extract those proteins whose levels were significantly altered in each of the studies. Thus, we have generated a panel of specific proteins that consistently appear in these studies to be over- or under-represented; and we detail their frequencies. Finally, as an important part of our study, we present a well-characterized and validated peptide analysis of all MS-data obtained from the proteins that show consistent changes (up- or down- regulation). A deep study of this novel AD peptide database, containing more than 3000 tryptic peptides, has led us to unveil a new panel consisting of the most commonly identified peptides in AD CSF, including the peptide direction change in each independent study; significance; and abundance in these clinical cohorts.

## Methods

### Data sources and search strategy

**Fig. 1:**
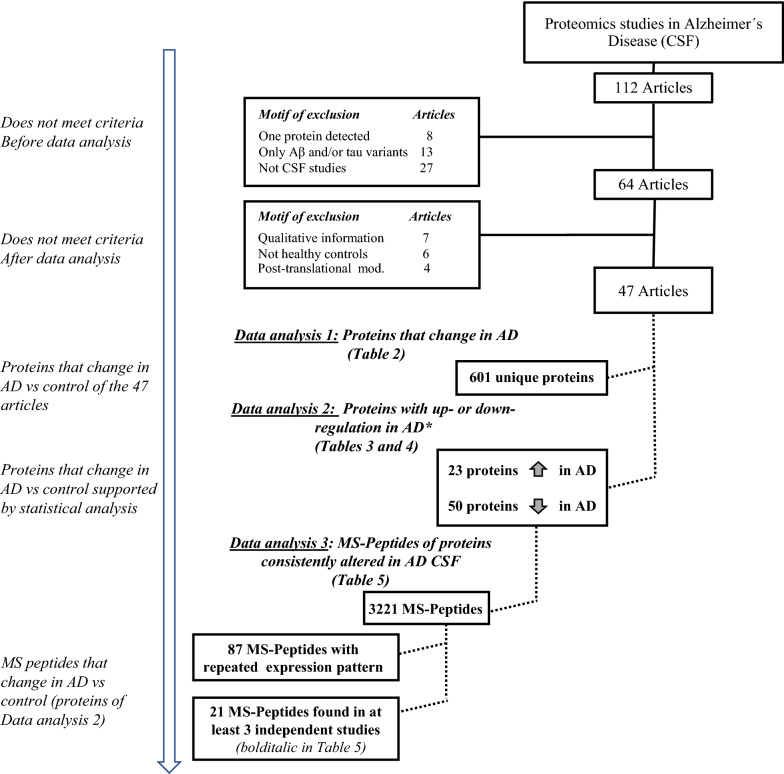
Methodological flow chart of the search strategy in the PubMed database. *Only those proteins that appeared consistently and statistically up- or down-regulated in AD in at least 2 studies were considered further

We searched MEDLINE (via PubMed) to December 2019. The keywords to perform the advanced search were “cerebrospinal fluid” and/or “CSF”, “proteomics”, “peptidomics” and “Alzheimer’s”. We also searched for additional terms such as “biomarkers” and for specific proteomic approaches (i.e. LC–MS/MS) (Fig. 1).

### Inclusion criteria and construction of the database

To strengthen the study, we included only studies that identified more than one protein as an AD biomarker in CSF, removing 8 studies that focused on only one protein (see Fig. 1) or those that only provided qualitative information (n = 7). We also removed those studies that involved only Aβ and tau variants/isoforms (n = 13); studies focused exclusively on post-translational modifications (PTM) (n = 4); and others that, although containing the search parameters within the text, did not actually perform experimental proteomics research on CSF (for example, in plasma samples) (n = 27) (Fig. 1).

Neuropsychological criteria for the definition of diagnostic groups were heterogeneous (Table 1). Baseline characteristics to define healthy controls according to cognitive criteria were: CDR = 0, no neuropsychological deficits and/or not reaching mild cognitive impairment (MCI) criteria; score ≥ 23 on Montreal Cognitive Assessment; MMSE score of 30; and asymptomatic NCs, mutation non-carriers. Baseline criteria to define AD were the presence of self-reported cognitive complaints; cognitive criteria were CDR ≥ 1; score < 23 on the Montreal Cognitive Assessment; subjects who met criteria for probable AD dementia based on NIA-AA criteria; and familial Alzheimer disease (FAD) mutation carriers (MCs). In both control and AD groups, 23 out of the 47 studies confirmed an AD diagnosis using the CSF biomarkers Aβ42, T-tau and/or P-tau, and provided clinical evaluations that included the following: detailed informant-based history, assessment of medical records, medical history, family history, physical and neurologic examination, routine lab tests, brain CT or MRI, brain imaging neurological and cognitive examinations. Additional details are shown in Table 1. Thus, we have not included studies that contain the following: categories of pre-MCI individuals or subjective memory complaints (SMC); articles comparing Alzheimer’s disease proteins with Parkinson’s disease proteins; articles discussing protein differences between younger and older subjects; and articles that studied proteins before and after a specific treatment (n = 6).

**Table 1:** Proteomic approaches, sample size and neuropsychological criteria for the groups used in the studies included

No	Author and year	Proteomic approach	Controls samples	AD samples	Mean age (controls)	Mean age (AD)	Neuropsychological criteria	CSF biomarkers (control)	CSF biomarkers (AD)
1	Whelan CD et al. (2019) [86]	Olink™ ProSeek immunoassay	565	176	72	74.6	NINCDS-ADRDA criteria, MMSE score and CSF biomarkers	Mean Aβ42 [pg/ml(SD)]: 752(253) Mean Aβ40 [pg/ml(SD)]: 5847(2042) Aβ42/40 ratio - log2 transformed (SD): 2.05(0.17) Mean total tau [pg/ml(SD)]: 292(89) Mean phospho-tau [pg/ml(SD)]: 37(13)	Mean Aβ42 [pg/ml(SD)]: 305(132) Mean Aβ40 [pg/ml(SD)]: 5470(2179) Aβ42/40 ratio - log2 transformed (SD): 2.9(0.29) Mean total tau [pg/ml(SD)]: 649(221) Mean phospho-tau [pg/ml(SD)]: 123(47)
2	Khoonsari et al. (2019) [87]	Nano LC-MS/MS (7 T hybrid LTQ FT MS)	45	76	88	72	NINCDS-ADRDA criteria, DSM-IV criteria, MMSE and CSF biomarkers	Aβ42 ng/l (Median[range]): 676(337–1343) p-tau ng/l (Median[range]) 414 (202–1121) t-tau ng/l (Median[range]): 63 (29-122)	Aβ42 ng/l (Median[range]): 405(160–1160) p-tau ng/l (Median[range]) 82 (28–220) t-tau ng/l (Median[range]): 617 (160–1720)
3	Sathe et al. [88]	High-Resolution MS, TMT multiplexing technology and targeted PRM analysis	5	5	71	71.6	CDR, MOCA, CSF biomarkers and NIA-AA criteria	Mean Aβ42 [pg/ml(SD)]: 1185.86(389.29)	Mean Aβ42 [pg/ml(SD)]: 402.11(189.62)
4	Duits et al. (2018) [89]	PRM	40	40	64.5	64.6	MMSE, clinical evaluation, and CSF biomarkers	Mean Aβ42 [pg/ml(SD)]: 1086(161) Mean total tau [pg/ml(SD)]: 228(64) Mean phospho-tau [pg/ml(SD)]: 40(9)	Mean Aβ42 [pg/ml(SD)]: 640(91) Mean total tau [pg/ml(SD)]: 740(433) Mean phospho-tau [pg/ml(SD)]: 94(47)
5	Dayon et al. (2018) [90]	RP LC-MS/MS	48	72	66	73.7	CDR, APOEa genotype and CSF biomarkers	Mean Aβ42 [pg/ml(SD)]: 957.4(194) Mean total tau [pg/ml(SD)]: 221.5(82.9) Mean phospho-tau [pg/ml(SD)]: 45.9(13.3) Mean phospho-tau (SD)/Aβ42: 0.049(0.015)	Mean Aβ42 [pg/ml(SD)]: 774(281.5) Mean total tau [pg/ml(SD)]: 471.1(316.6) Mean phospho-tau [pg/ml(SD)]: 72.7(40.9) Mean phospho-tau (SD)/Aβ42: 0.114(0.097)
6	Brinkmalm et al. (2018) [14]	PRM-MS analysis and previously nanoflow LC-MS/MS	15	10	64.9	62.7	Clinical evaluation, computer tomography (CT) scan and CSF Aβ42	Mean Aβ42 [pg/ml(SD)]: 877.2(206.3)	Aβ42 < 400 pg/ml;Mean Aβ42 [pg/ml(SD)]: 275.4(106.8)
7	Skillbäck et al. (2017) [45]	Quantification-driven proteomic	40	40	64.5	64	NIA and IWG-2 criteria, CSF biomarkers and MMSE	Information extracted from graphs: Mean Aβ42 [pg/ml]: 950 Mean total tau [pg/ml]: 260 Mean phospho- tau [pg/ml]: 55	Information extracted from graphs: Mean Aβ42 [pg/ml]: 450 Mean total tau [pg/ml]: 600 Mean phospho- tau [pg/ml]: 85
8	Wang et al. (2016) [46]	LC-MS/MS and lectin-enrichment chromatography	4	4	74	75.7	Neuropsychological tests	NI	
9	Paterson et al. (2016) [91]	Targeted proteomics: MRM-based triple quadrupole mass spectral assay	36	46	58.5	62.9	APOE^#^ genotype and CSF biomarkers	Mean Aβ42 [pg/ml(SD)]: 960(291) Mean total tau (range): 234.5(174.5–315.5) Mean phospho-tau [pg/ml(SD)]: 35.5(13.2) Mean phospho-tau (range)/Aβ42: 0.25(0.19-0.33)	Mean Aβ42 [pg/ml(SD)]: 408(2168) Mean total tau (range): 947(760–1196-315.5) Mean phospho-tau [pg/ml(SD)]: 107.5(38.12) Mean phospho-tau (range)/Aβ42: 2.5(1.8-4.1)
10	Remnestal et al. (2016) [92]	Suspension bead array: HPA antibodies targeting the 280 proteins	38	72	58	77.4	NINCDS-ADRDA criteria and CSF biomarkers	Mean Aβ42 [pg/ml(range)]: 706(559-1192) Mean total tau (range): 308(171-399) Mean phospho-tau (range): 47(29-60)	Group AD1: Mean Aβ42 [pg/ml(range)]: 350(160-950) Mean total tau (range): 600(210–2430) Mean phospho-tau (range): 78(34–282)Group AD2: Mean Aβ42 [pg/ml(range)]: 453(260–639) Mean total tau (range): 834(190–3178) Mean phospho-tau (range): 86(59–179)
11	Khoonsari et al. (2016) [16]	RP nano LC- MS/MS andantibody suspension bead arrrays	10	10	88.2	79.4	Clinical evaluation, CSF biomarkers and MMSE	NI	Mean Aβ42 [ng/l]: 420.1 Mean total tau [ng/l]: 652.1 Mean phospho-tau [ng/l]: 132.2
12	Domenico et al. (2016) [93]**	2-DE - WB - MS (2-DE gel/carbonyl immunoblot)	6	6	NI	NI	NINCDS-ADRDA criteria, neuropsychological test and APOE^#^ genotype	NI	
13	Heywood et al. (2015) [94]	Label-free proteomic analyses (2-DE LC-MS) and Targeted proteomics: MRM-based triple quadrupole mass spectral assay	15	16	61	68.1	CSF biomarkers	Mean Aβ42 [pg/ml(SD)]: 905.41(237) Mean total tau [pg/ml(SD)]:136.58(162) Mean phospho-tau [pg/ml(SD)]: 19.33(11.11)	Mean Aβ42 [pg/ml(SD)]: 503.68(165.8) Mean total tau [pg/ml(SD)]:733.77(481.25) Mean phospho-tau [pg/ml(SD)]: 93.38(31.55)
14	Hendrickson et al. (2015) [20]	RP nano-HPLC coupled to an LTQ-FTMS hybrid mass spectrometer. SRM-MS	30	30	69	70	NINCDS-ADRDA criteria and APOE^#^ genotype	NI	
15	Spellman et al. (2015) [95]	LC/MRM-MS	85	66	75.6	75	APOE^#^ genotype, CSF biomarkers and MMSE	Mean Aβ42 [SD]: 207.8(56.26) Mean phospho-tau (SD): 24.19(12.02)	Mean Aβ42 [SD]: 141.12(37.39) Mean phospho-tau (SD): 41.95(20.6)
16	Hölttä et al. (2015) [15]	TMT, LC−ESI MS, LC−MALDI MS	8	8	65	77	DSM-IIIRand NINCDS-ADRDA criteria	Mean Aβ42 [pg/ml(range)]: 700(545–817) Mean total tau (range): 405(330–523)	Mean Aβ42 [pg/ml(range)]: 235(210–298) Mean total tau (range): 800(717–925)
17	Leung et al. (2015) [96]	MAP-RBM platform (XMAP Luminex platform) /ELISA	325	344	71.7	74	MMSE, CDR, NINDS-ADRDA criteria and CSF biomarkers	Aβ42 > 192 pg/ml	Aβ42 < 192 pg/ml
18	Khan et al. (2015) [97]**	XMAP Luminex platform	88	65	75.8	74.6	APOE^#^ genotype, CSF biomarkers, and MMSE	Mean Aβ42 [(SD)]: 205.7(57.2) Mean total tau (SD):69.2(27.9) Mean phospho-tau (SD): 24.9(13.2)	Mean Aβ42 [(SD)]: 140.4(35.3) Mean total tau (SD):125.9(60.3) Mean phospho-tau (SD): 42.2(20.7)
19	Oláh et al. (2015) [98]	Master Antibody Microarray and WB	25	25	74.5	72	Clinial evaluation, NINCDS-ADRDA criteria, APOE^#^ genotype and CSF biomarkers	Mean Aβ42 [pg/ml(SD)]: 794(20) Mean total tau [pg/ml(SD)]: 341(171) Mean phospho-tau [pg/ml(SD)]: 23(2)	Aβ42 < 500 pg/ml total tau >600 pg/ml phospho-tau>60 pg/ml
20	Alzate et al. (2014) [99]	MS and MS/MS and 2-DE multiplexed WB	7	11	AR: 62.8 to 81.5	AR: 62.8 to 81.5	APOE^#^ genotype	NI	
21	Wildsmith et al. (2014)[100]	LC-MS/MS, Targeted LC-MRM and ELISA	10	45	68.5	77.5	MMSE and CSF biomarkers	Information extracted from graphs: Mean Aβ42 [pg/ml]: 650 Mean total tau [pg/ml]: 60 Mean phospho- tau [pg/ml]: 25	Information extracted from graphs: Mean Aβ42 [pg/ml]: 400 Mean total tau [pg/ml]: 150Mean phospho- tau [pg/ml]: 60
22	Chakrabarti et al. (2014) [101]	2-DE, MALDI MS Analysis and co-immunoprecipitation	11	11	67	71	NINCDS-ADRDA criteria, DSM-IV criteria, MMSE, CDR and other tests	NI	
23	Choi et al. (2013) [49]**	Nano LC-MS/MS and nano LC-MRM/MS and ELISA	3	3	NI	NI	Confirmed by autopsy	NI	
24	Wijte et al. (2012) [102]	MALDI TOF/TOF MS	20	20	77.7	75.95	Postmortem neuropathological evaluation	NI	
25	Ringman et al. (2012) [103]	MS/MS, 2-DE LC/MS	5	4	37.6	34.2	CDR and FAD mutation carriers (MCs) and related noncarrieres (NCs)^Π^. APOE^#^ genotype and CSF biomarkers	Mean Aβ42 [pg/ml(SD)]: 618.4(100.1) Mean total tau [pg/ml(SD)]: 50.5(9.4) Mean phospho-tau [pg/ml(SD)]: 24.6(9.2)	Mean Aβ42 [pg/ml(SD)]: 277.1(162.9) Mean total tau [pg/ml(SD)]: 140(59.9) Mean phospho-tau [pg/ml(SD)]: 67(29)
26	Manral et al. (2012) [104]	2-DE and Nano LC-ESI-Q-TOF MS/MS system	8	8	60.7	62.6	NINCDS-ADRDA criteria, MMSE score and MRI	NI	
27	Vafadar-Isfahani et al. (2012) [105]	nLC-MALDI-TOF MS/MS and MALDI TOF/TOF MS	23	33	NI	NI	NINCDS-ADRDA criteria, DSM-IV and MMSE and clinical evaluation	Information extracted from graphs: Mean Aβ42 [pg/ml]: 440 Mean Aβ40 [pg/ml]: 5000 Mean total tau [pg/ml]: 500	Information extracted from graphs: Mean Aβ42 [pg/ml]: 190 Mean Aβ40 [pg/ml]: 3900 Mean total tau [pg/ml]: 2000
28	Jahn et al. (2011) [44]	CE-MS and MS/MS	17	34	58.9	69	NINCDS-ADRDA criteria, CSF biomarkers and MMSE score	NI	Mean Aβ42 [pg/ml]: 300.91 Mean total tau [pg/ml]: 541.21 Mean phospho-tau [pg/ml]: 81.84
29	Craig-Schapiro et al. (2011) [106]**	ELISA (targeted)	242	28+63	71.6	76.8	NINCDS-ADRDA criteria and CDR	NI	
30	Perrin et al. (2011) [50]	2-DE DIGE / nano- LC-MS/MS	198	29	71	76.5	CDR, MMSE score and CSF biomarkers	Mean Aβ42 [pg/ml(SD)]: 605(240) Mean total tau [pg/ml(SD)]: 304(161) Mean phospho-tau [pg/ml(SD)]: 55(25)	Mean Aβ42 [pg/ml(SD)]: 351(118) Mean total tau [pg/ml(SD)]: 552(263) Mean phospho-tau [pg/ml(SD)]: 77(38)
31	Hu et al. (2010) [107]**	XMAP Luminex platform	33	66	NI	NI	MMSE, CSF biomarkers and confirmed by autopsy	NI	
32	Maarouf et al. (2009) [108]	2-DE DIGE and MS	43	47	84	79	APOE^#^ genotype, CERAD and the NIA-Reagan Institute	NI	
33	Yin et al. (2009) [109]	1-DE and LC–MS/MS	6	5	55.3	71	NINCDS-ADRDA criteria	NI	
34	Zhang et al. (2008) [110]	Immunobead-based multiplex assays	95	48	63	70	Clinical evaluation	NI	
35	Jung et al. (2008) [66]	2-DE, DE-MALDI-TOF MS	30	27	73.2	77.7	NINCDS-ADRDA criteria and CDR	NI	
36	Simonsen et al. (2007) [111]	MS	32	85	68.3	69.15	NINCDS-ADRDA criteria, MMSE score and clinical evaluation	NI	
37	Hu et al. (2007) [112]**	2-DE DIGE, MS/MS and ELISA	55	19	NI	NI	CSF biomarkers, CDR and clinical evaluation	Information extracted from graphs: Mean Aβ42 (range): 1(0–2) Mean total tau (range): 0.9(0–5)Mean phospho- tau (range): 0.8(0–5)	Information extracted from graphs: Mean Aβ42 (range): 0.6(0–2) Mean total tau (range): 1.9(0–5)Mean phospho- tau (range): 1.1(0–5)
38	Korolainen et al. (2007) [113]	2-DE and Nano LC-MS/MS	8	11	64.7	73.54	APOE^#^ genotype, NINCDS-ADRDA criteria and MMSE score	NI	
39	Finehout et al. (2007) [114]	2-DE and TOF/TOF MS	12	44	NI	NI	Postmortem confirmation	NI	
40	Simonsen et al. (2008) [115]	SELDI-TOF MS	72	95	73.1	72.5	NINCDS-ADRDA criteria, MMSE score and CSF biomarkers	Group C1: Mean Aβ42 [pg/ml(SD)]: 876(209.2) Mean total tau (SD): 295.8 (153.6) Group C2: Mean Aβ42 [pg/ml(SD)]: 748.9(155.3) Mean total tau (SD): 332.9 (158.7)	Group AD1: Mean Aβ42 [pg/ml(SD)]: 524.6(170.1) Mean total tau (SD): 739.1 (300.9) Group AD2: Mean Aβ42 [pg/ml(SD)]: 464(187.7) Mean total tau (SD): 657.7 (339.4)
41	Castaño et al. (2006) [116]	2-DE and MALDI-TOF MS	43	43	81	81	Clinical diagnosis, Braak stages, and CERAD	NI	
42	Abdi et al. (2006) [52]	iTRAQ labeling and 2-DE LC, MS/MS and WB	10	10	67	72	MMSE, CDR, NINDS-ADRDA criteria and other test	NI	
43	Selle et al. (2005) [117]	MALDI-TOF-MS	102	140	62.5	71	ICD-10, NINCDS-ADRDA criteria, DSM-IV and MMSE	NI	
44	Zhang et al. (2005) [118]**	LC-MS ICAT	20	32	70.5	71	MMSE and CDR	NI	
45	Puchades et al. (2003) [68]	2-DE and MALDI-TOF MS	7	7	66	80	NINCDS-ADRDA criteria and MMSE score	NI	
46	Carrette et al. (2003) [119]	SELDI-TOF MS using SAX2 Protein-chip arrays	10	19	78	75	NINCDS-ADRDA criteria	NI	
47	Davidsson et al. (2002) [120]	2-DE, SYPRO Ruby staining and MS	12	15	67.3	77.2	NINCDS-ADRDA criteria and MMSE score	NI	
			Total samples (Control)	Total samples (AD)	Average age (Control)	Average age (AD)			
			2562	2022	70	73			

*ESI* electrospray ionization, *MRM* multiple reaction monitoring, *PRM* parallel reaction monitoring, *MS* mass spectrometry, *TMT* tandem mass tag, *RP-LC MS/MS* reversed-phase LC-MS/MS, *SRM* reaction monitoring, *CE-MS* capillary electrophoresis mass spectrometry, *SELDI-TOF-MS* surface enhanced laser desorption/ionization time-of-flight mass spectrometry, *WB* Western Blot, *AR* age range, *NI* not information, *CDR* Clinical dementia rating scale, *MOCA* Montreal cognitive assessment, *NINCDS-ADRDA* National Institute of Neurological and Communicative Disorders and Stroke and the AD and Related Disorders Association, *MMSE* Mini-menta state exam, *DSM* Diagnostic and Statistical, *FAD* familial Alzheimer Disease, *CERAD* Consortium to Establish a Registry for Alzheimer’s Disease, *ICD-10* 10th revision of the International Statistical Classification of Diseases and Related Health Problems, *IWG-2*  International Working Group-2 criteria

^#^Studies based on APOE genotype

^Π^Study based on PSEN1 and APP mutations (this study was not considered to calculate the average age)

** Qualitative and not quantitative data

We examined both tables and information included in the main texts, as well as associated supplemental data, to extract information on protein and peptide sequences that were found to be altered in the CSF of AD samples. However, we included in our analysis only those articles that provided statistics for their results by showing significant differences between control and AD groups through numerous statistical methods such as the non-parametric Mann–Whitney U and Kruskal–Wallis tests, which were the most common statistical approaches among the different studies [14–16]. To solve the problem of heterogeneity in the annotation databases used in the various studies, we made all protein names consistent with the Uniprot database (https://www.uniprot.org) by filtering them through Ingenuity Pathway Analysis (IPA, http://www.ingenuity.com) and DAVID bioinformatics resources (http://david.abcc.ncifcrf.gov.), which enabled us to obtain the Entrez Gene ID of all proteins. To do this, it has been necessary to remove “hypothetical proteins”, “unidentified proteins”, “IgG light chain” “IgG heavy chain” and “predicted protein”. Variants of amyloid-β and tau, the focus of several reviews [10, 17] were not included. Of note, the FLJ00385 protein and all proteins depicted with Δ were not recognized by DAVID bioinformatics resources.

### Data mining

We included in our analysis exclusively those studies which contained quantitative protein information, according to each study-specific cut-off point with a given statistical significance. The protein database, filtered as described above, was first analyzed to identify the unique proteins that change between AD and control samples. To analyze all proteins, we extracted related information based on different annotation aspects/terms which was inconsistent in the different studies. For example, Apolipoprotein J, APOJ, APO-J, CLUS_HUMAN, P10909, and TRPM-2 all are referred to Clusterin.

After generating a table containing all proteins that exhibit changes in AD (Additional file 1: Table S1), we calculated the number of articles that identify each specific protein and we further considered those proteins that appeared in 2 or more studies to generate a table that indicated whether they were found to increase or decrease in the context of AD.

An important part of our study consisted in the direct analysis of all MS-data to generate a database of all peptides corresponding to proteins with consistent changes (up- or down- regulation). This database includes all peptide sequences extracted from the proteins mentioned above, the observed peptide mass weight (Da), the significance difference statistics, the ratio of AD/Control or fold-change, and the direction change in each article. It should be noted that the significance of certain peptide changes had not been included in all studies, and so we independently calculated this parameter from raw data using paired t-tests. In addition, the peptide masses were calculated using the pI/Mw tool (https://web.expasy.org/compute_pi/). We considered only those peptides that appeared at least in two independent studies and analyzed whether their changes were supported by statistical analysis; and those observed in 3 or more independent studies were considered for discussion purposes as the most consistently observed in the context of AD.

### Pathway analysis

Pathway, network and upstream regulator analysis were generated through the use of IPA (QIAGEN Inc., https://www.qiagenbio-informatics.com/products/ingenuity-pathway-analysis) with the proteins obtained in the current study, taking into consideration whether they increased or decreased in the context of AD. The threshold for the top canonical pathways was increased to ¡–Log (p value) 4.5, and only the most relevant network of proteins was considered. Upstream regulators were also investigated, not considering non-endogenous chemical drugs and toxicants. A bias correction of the z-score was performed and activation z-scores below 2 were not further considered in order to reduce artifactual results.

### Risk of bias assessment

We have combined all of the statistical studies for comparative purposes, although the varying degrees of statistical significance intrinsic to the different proteomics and peptidomics approaches must be considered. Although in this study four independent investigators conducted literature searches to identify all possible protein data, we encountered difficulties in extracting the information within several articles, as the annotation datasets used differed between articles, and the statistical approaches were sometimes not clearly described. To allow the reader access to the original extracted information of proteins, we have included Additional file 1: Table S1.

As for the peptide database, it is important to note that only 17 articles reported actual peptide sequences; thus, this peptide analysis constitutes a different analysis than the one carried out with proteins.

## Results

Our initial bibliographic screens support the idea that, among the different pathologies that affect the brain, AD is likely the most investigated from a proteomics point of view, with a total of 112 proteomics-related articles (Fig. 1). In order to generate a reliable database with only those studies that contain statistically significant proteomic data between AD and control samples- controls being exclusively healthy individuals (see Table 1 for information regarding neurophysiological criteria), we did not consider studies/data specific to MCI. After this screening, 47 articles were included in our analyses (Fig. 1). From these 47 articles, we generated a database that contains proteomic studies from a total of 2022 AD patients with a mean age of 73 years and a total of 2562 samples of healthy individuals with a mean age of 70 years (Table 1). Annotation databases used in the different articles to identify the proteins differed; therefore, a refinement of the database was needed to generate similar data outputs using IPA and DAVID bioinformatic resources. Information on the meaning of the abbreviations used for each protein and article (numbered from 1 to 47) is shown in Additional file 1: Table S1.

### Analysis of the proteins that were found to change in AD *vs* control samples

Additional file 2: Table S2 shows information related to the proteins identified as changing in AD, along with the number of articles that found changes in a particular protein. After matching the proteomic information between the different articles, a total of 601 unique proteins were identified (Additional file 2: Table S2). The proteins that appeared in at least 2 proteomic studies are shown in Table 2 (162 proteins). The most recurrent proteins were Apolipoprotein E (APOE), Nerve growth factor inducible (VGF) and Transthyretin (TTHY), being identified in 18, 15 and 14 independent proteomics studies, respectively (Table 2). In order to provide information on the faddabundance of each protein, the 50 most abundant proteins in the CSF of healthy individuals, according to published sources [18], are designated with $ in Tables 2, 3 and 4.

**Table 2:** Proteins that show differences between AD and control groups across independent studies

Number of articles	Gene (Protein)
18	*APOE* (APOE)^$^
15	*VGF* (VGF)
14	*TTR* (TTHY)^$^
11	*CHGA* (CMGA)^$^, CST3 (CYTC)^$^
10	*SERPINA1* (A1AT)^$^, APOA1 (APOA1)^$^
9	*C4* (CO4)^$^, *CLU* (CLUS)^$^, *SPP1* (OSTP)^$^, *PCSK1N* (PCSK1N)
8	*C3* (CO3)^$^
7	*PTGDS* (PTGDS)^$^, B2M (B2MG), GC (VTDB)^$^
6	*ALB* (ALBU)^$^, *A2M* (A2MG)^$^, *APOH* (APOH)^$^, *CHI3L1* (CH3L1), *NPTX1* (NPTX1), *NPTXR* (NPTXR), *SCG2* (SCG2), *RBP4* (RET4)
5	*TF* (TRFE)^$^, *AGT* (ANGT)^$^, *FABP* (FABPH), *GSN* (GELS)^$^, *HP* (HPT)^$^, *SERPINA3* (AACT)^$^, *CNDP1* (CNDP1)^$^, *CP* (CERU)^$^, *CHGB* (SCG1)^$^, *ITM2B* (ITM2B), *SCG3* (SCG3)
4	*A1BG* (A1BG)^$^, *FGB* (FIBB), *PLG* (PLMN)^$^, *NRXN1* (NRX1A), *MDH1* (MDHC), *APOD* (APOD)^$^, *CFB* (CFAB)^$^, *HPX* (HEMO)^$^, *IGFBP2* (IBP2), *RETN* (RETN), *DAG1* (DAG1), *APP* (A4), *FGA* (FIBA), *SPRC* (SPARC), *SPON1* (SPON1), *GOT1* (AATC), *APLP2* (APLP2), *SCG5* (7B2), *CLSTN1* (CSTN1), *MCAM* (MUC18), SERPINF1 (PEDF)^$^
3	*AMBP* (AMBP), *ITIH1* (ITIH1), *LRG1* (A2GL), *MT3* (MT3), *NEGR1* (NEGR1), *RNASE1* (RNASE1), *SORT1* (SORT), *APOC1* (APOC1), *ALDOA* (ALDOA), *C2* (CO2), *PKM* (KPYM), *AFM* (AFAM), *APOA2* (APOA2)^$^, *CLEC3B* (TETN), *CFD* (CFAD), *HGF* (HGF), *PPY* (PAHO), *PSAP* (SAP),*VEGF* (Q9UNS8), *CNTN1* (CNTN1), *NCAM1* (NCAM1), *SPARCL1* (SPRL1)^$^, *CXCL16* (CXL16), *SPOCK1* (TICN1), *VEGFA* (VEGFA), *EFEMP1* (FBLN3)^$^, *BASP1* (BASP1), *KNG1* (KNG1)^$^, *NSG1* (NSG1), *PRNP* (PRIO), *SERPINC1* (ANT3)^$^, *SMOC1* (SMOC1), *SOD1* (SODC), *APLP1* (APLP1)
2	*CTSD* (CATD), *ACTA2* (ACTA), *ATP6AP1* (VAS1), *AZGP1* (ZA2G)^$^, *C1QB* (C1QB), *C5* (CO5), *C6* (CO6), *ENO2* (ENOG), *ENPP2* (ENPP2)^$^, *IGF2* (IGF2), *KLK6* (KLK6)^$^, *LCAT* (LCAT), *LTBP2* (LTBP2), *LYNX1* (LYNX1), *NFASC* (NFASC), *NRGN* (NEUG), *NRXN3* (NRX3B), *PAM* (AMD), *PPIB* (PPIB), *PTPRD* (PTPRD), *PTPRN* (PTPRN), *SEZ6L* (SE6L1), *TAC1* (TKN1), *GAP43* (NEUM), *CHL1* (NCHL1)^$^, *ORM1* (A1AG1)^$^, *KRT9* (K1C9), *IGFBP7* (IBP7), *LDLR* (LDLR), *MMP10* (MMP10), *TNFSF10* (TNF10), *ADIPOQ1* (ADIPO), *CPE* (CBPE), *CCL16* (CCL16), *CD14* (CD14), *CD40* (TNR5), *C7* (CO7), *C1R* (C1R), *DCD* (DCD), *HRG* (HRG), *IGFBP6* (IBP6), *INS* (A6XGL2), *L1CAM* (L1CAM), *MMP2* (MMP2), *MB* (MYG), *NBL1* (NBL1), *NPDC1* (NPDC1), *NRCAM* (NRCAM)^$^, *OGN* (MIME), *PRL* (Q5THQ0), *SERPINA7* (THBG), *SERPINE1* (PAI1), *CNTN2* (CNTN2), *CD99* (CD99), *ITM2C* (ITM2C), *PENK (*PENK), *SPOCK2* (TICN2), *STT* (SMS), *TAC* (TKNK), *ITIH4* (ITIH4), *SOD3* (SODE), *VTN* (VTNC), *CADM3* (CADM3), *CLSTN3* (CSTN3), *COL1A1* (CO1A1), *FBLN1* (FBLN1)^$^, *IGSF8* (IGSF8), *NCAN* (NCAN), *PBXIP1* (PBIP1), *PGLYRP2* (PGRP2), *TGOLN2* (TGON2)

The number of studies which detected these changes is included. **$** proteins which are abundant in CSF of healthy individuals (Schilde et al. 2018)

**Table 3:**
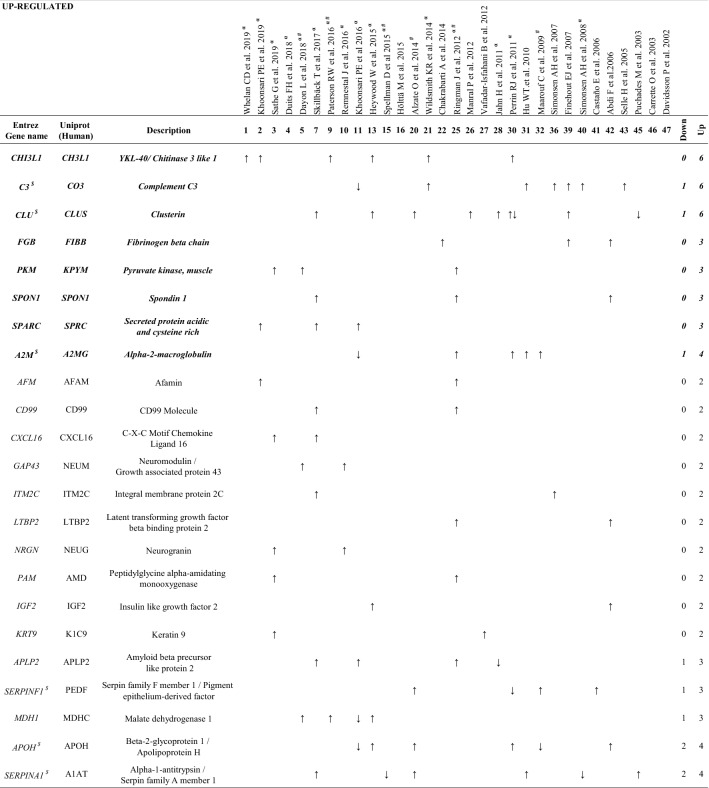
Proteins increased in AD CSF proteomic studies

Proteins with the same pattern of expression (toward the same direction) at least in 2 or 3 (bolditalic proteins) independent studies

^$^Proteins abundant in CSF of healthy individuals (Schilde et al. 2018)

^α^Studies that provide Aβ and Tau contents in CSF

^#^APOE genotype to diagnose AD

**Table 4:**
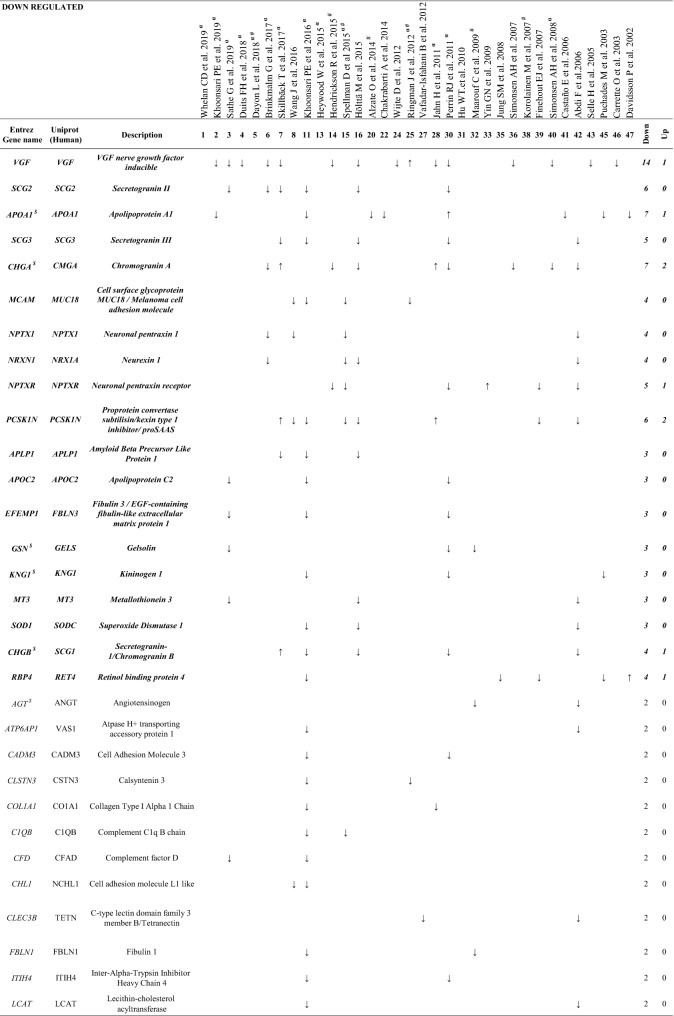

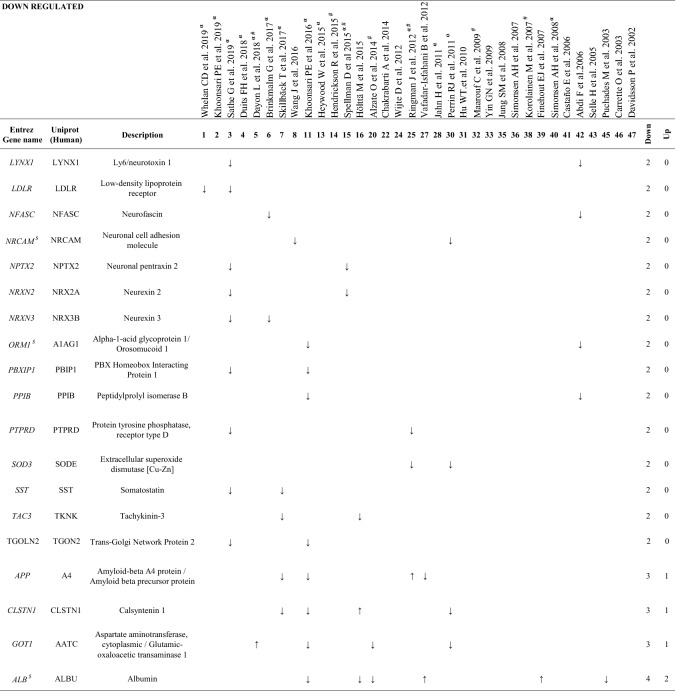
Proteins decreased in AD CSF proteomic studies

Proteins with the same pattern of expression (toward the same direction) at least in 2 or 3 (bolditalic proteins) independent studies

^$^Proteins abundant in CSF of healthy individuals (Schilde et al. 2018)

^α^Studies that provide Aβ and Tau contents in CSF

^#^APOE genotype to diagnose AD

We found that, of the proteins that were identified in more than one study, 63 proteins appeared both as increased and decreased in different proteomic studies and therefore were considered as inconsistent (Additional file 3: Table S3). On the other hand, we identified 23 proteins which were increased (Table 3), and 50 proteins decreased (Table 4) in AD samples in at least in 2 independent studies. Of these, a set of 27 proteins, indicated in bolditalic in Tables 3 and 4, represents the most consistent findings across the proteomic literature, being observed with the same pattern of expression at least in 3 independent studies. It is interesting to point out that although some of the studies included in our analysis used depletion kits prior to proteomics, from all possible proteins affected by the use of these kits, only 2, Albumin (ALB) and Fibrinogen beta chain (FIBB), were among the proteins detected in our study.

**Fig. 2:**
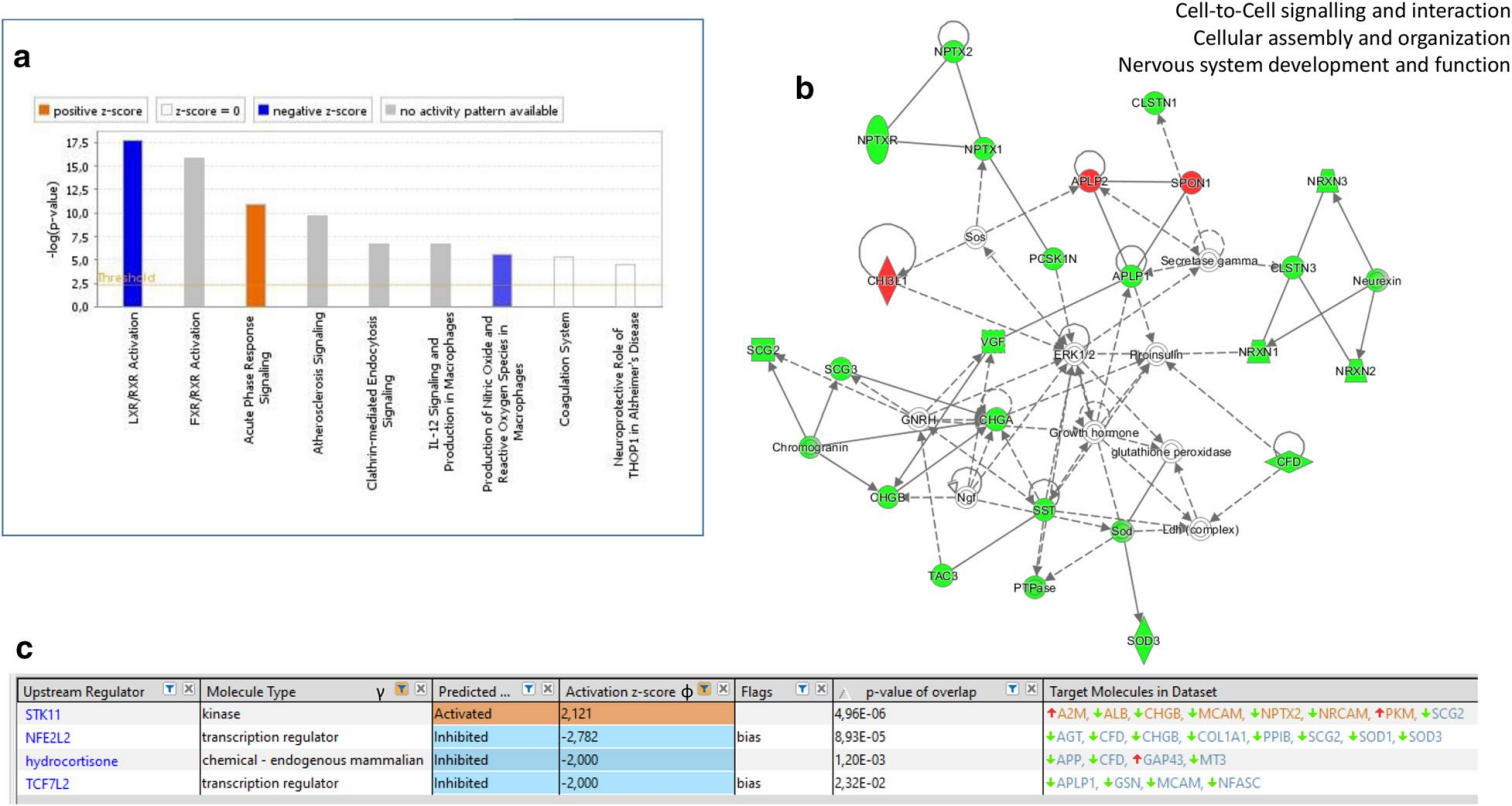
Pathways, network and upstream regulators analysis of the proteins increased or decreased in AD. **a** Canonical pathways were analyzed using IPA. The threshold for the top canonical pathways was set to –Log (p value) 4.5, and positive and negative z-scores are shown. **b** The most consistent protein network corresponds to cell-to-cell signalling-cellular assembly and organization-nervous system development and function. **c** Upstream regulators were investigated. γ, non-endogenous chemical drugs and toxicants were excluded. φ activation z-score was increased to 2. *bias* correction of the z-score is indicated. Green, proteins decreased in AD. Red, Proteins increased in AD

A pathway analysis of the subset of proteins exhibiting consistent changes (Tables 3 and 4) was performed using IPA, taking into account the significance of their increase or decrease. LRX/RXR activation appeared as the pathway with the most members of the study along with a significant negative z-score (Fig. 2a). Additionally, one major interacting network was identified, related to cell-to-cell signalling: cellular assembly and organization-nervous system development and function (Fig. 2b). Our IPA analysis revealed that the transcription factor NFE2L2/NRF2 (nuclear factor, erythroid 2-like 2; predicted to be inhibited) and the Serine/Threonine Kinase 11 (STK11 kinase; predicted to be activated) were the most consistent potential upstream regulators (both z-scores > 2; P value < 0.0001; Fig. 2c).

### Analysis of the tryptic peptides of the proteins that change in AD *vs* control samples

In order to identify changes at the peptide level, we performed a direct analysis of MS-data of the 27 proteins consistently recognized as altered in AD. Information regarding peptide masses of these proteins was available in 17 studies. With the information extracted from these articles we were able to generate a database with 3221 peptide sequences (Additional file 4: Table S4). Further analysis of this database revealed that 87 peptides [Table 5 and Additional file 6: Figure S1 (sequences in bolditalic and in red)], which correspond to a total of 13 proteins (2 proteins increased and 11 decreased in AD) maintained a direction change consistent with that observed by proteomics in relation to the AD pathology (Table 5). Other peptides from these proteins showed inconsistent distribution across the different studies (Additional file 5: Table S5). On the other hand, 21 peptides (Table 5 (bolditalic) and Additional file 6 Figure S1 (underlined)] were found in at least 3 independent studies. These peptides correspond to Chitinase 3 like 1 (CH3L1, 2 peptides), VGF (9 peptides), Secretogranin-2 (SCG2, 1 peptide), ProSAAS (PCSKN1, 6 peptides), EGF-containing fibulin-like extracellular matrix protein 1 (FBLN3, 1 peptide), and Apolipoprotein C2 (APOC2, 2 peptides). Interestingly, CH3L1 was the only protein with no inconsistency in all identified peptides, since all were found to increase in AD (Table 5).

**Table 5:**
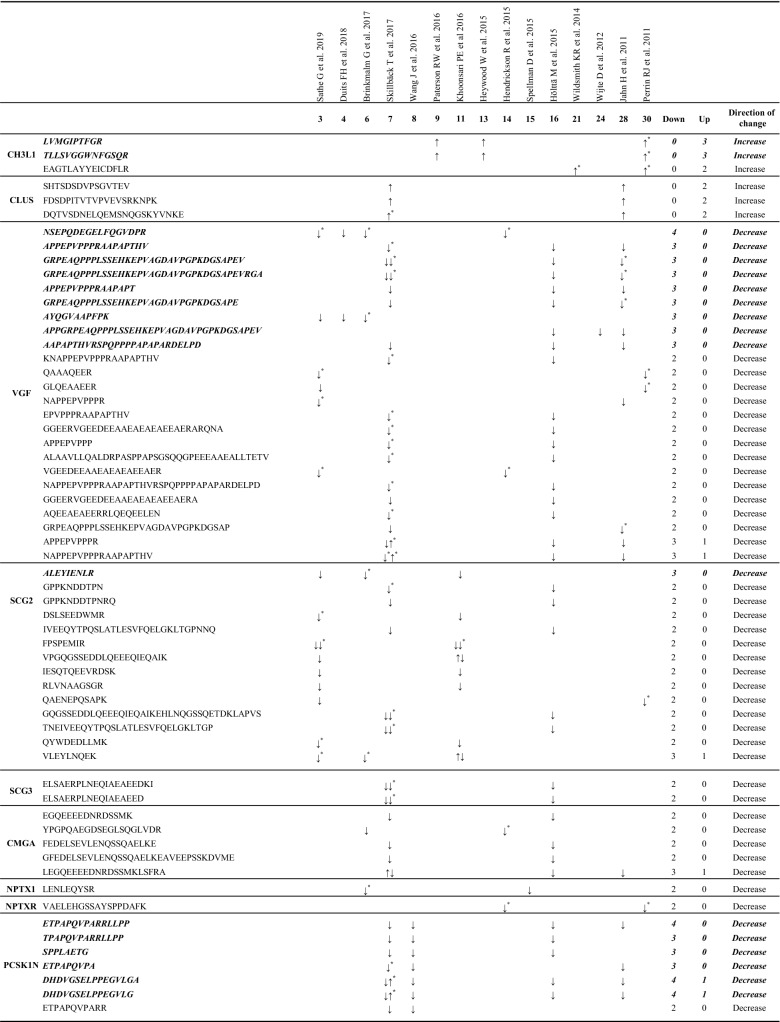

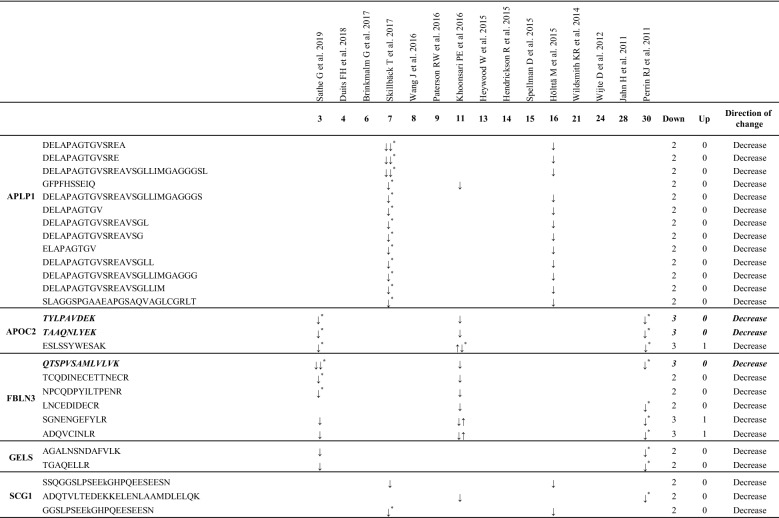
Peptide sequences from the proteins altered in AD CSF proteomic studies

Peptide sequence with the same pattern of expression (toward the same direction) at least in 2 or 3 (bolditalic proteins) independent studies

^*^Peptide sequences identified which show changes between AD and control samples supported by statistical analysis

## Discussion

In this comparative analysis we have compiled data from proteomic studies performed on human CSF samples obtained from AD and healthy individuals in order to construct a database of proteins and peptides that constitute the most reliable CSF biomarkers associated with an AD diagnosis. When comparing our hit proteins with the recently published CSF proteomics analysis performed by Wesenhagen et al., numerous differences are evident. An important distinction may be the use of different inclusion criteria in the two analyses. In order to generate a more accurate and reliable database, we exclusively took into consideration those proteins which were shown to be statistically significantly different between AD and control samples, according to each study-specific cut-off point. Therefore, our analysis relies exclusively on the basis of significant data, including hit distribution, direction change and significance, which could account for the discrepancies observed with Wesenhagen et al. We have also generated a novel database of peptides according to the proteomic findings that reveals the most consistently altered tryptic peptide biomarkers of AD within a given protein.

We initially identified APOE, TTHY, Osteopontin (OSTP) and Cystatin-C (CYTC) which are among the most abundant proteins in normal CSF [18] as the protein species whose levels change the most often in the context of AD. However, these 5 proteins appear both as increased and decreased in different proteomic studies. Although the number of studies included in Wesenhagen analysis was lower than ours, these findings are in agreement with their study [13], and prompts us to suggest that they cannot be considered as reliable CSF biomarkers of AD. While it is likely that heterogeneity in response direction may reflect irrelevant physiological or environmental factors, it is also interesting to speculate that this heterogeneity reflects unknown endophenotypes in AD. It is also possible that the changes in these 5 proteins provide an indication of the profound general protein dysregulation that occurs during AD progression. Additionally, our study reveals new information regarding proteins consistently altered in AD, including SPARC (SPRC), Kininogen-1 (KNG1) or Cell surface glycoprotein MUC18 (MUC18), whose role as potential CSF biomarkers for AD has not been investigated. These proteins are discussed below.

Among the most consistently down- and up-regulated AD proteomic biomarkers, our hit proteins with the highest occurrence are VGF and CH3L1, respectively. Both can be considered high quality CSF biomarkers, as they represent abundant proteins which consistently change in the same direction in 14 and 6 AD proteomics studies, respectively. VGF is a member of the granin family of proteins [19] previously proposed as a good marker for AD [10, 13], and a decrease in VGF-derived peptides in AD has been confirmed using other experimental approaches [14, 20, 21]. Interestingly, the 9 peptides that appear as reliable markers for the VGF down-regulation in AD correspond to both N- and C-terminal sequences, suggesting that the synthesis of the precursor protein itself is repressed in AD and not a specific derived peptide.

The levels of CH3L1, encoded by the *CHI3L1* gene, were also found to be increased in all 6 proteomics studies in which it was identified, just as reported by Wesenhagen et al. [22]. We propose that CH3L1, which is expressed in the CNS by microglia and astrocytes, constitutes one of the most interesting potential biomarkers, mainly because its physiological role in brain remains speculative. Increased expression of CH3L1 is found in human brains from pathologically confirmed AD individuals, implicating CH3L1 in the neuroinflammatory response to Aβ deposition [23]. Moreover, 2 independent ELISA studies, conducted to evaluate the role of CH3L1 as a CSF biomarker, have confirmed the observation of increased levels of CH3L1 in AD patients [24, 25]. Given that CH3L1 has been proposed as a common neuroinflammatory biomarker of other neurodegenerative diseases, [26, 27], the fact that 2 peptides (see Table 5) were consistently changed in AD studies assumes increased relevance. Although the presence of these peptides may arise as a consequence of intrinsic properties which render them highly identifiable by mass spectrometry, further characterization of these 2 peptides as AD biomarkers among other neurodegenerative diseases deserves investigation.

### Proteins consistently increased in AD CSF

In addition to CH3L1, other proteins were observed to increase in CSF samples from AD patients. Among those that stand out due to their consistency within the various studies (bolditalic proteins in Table 3) we found Complement C3 (CO3, 6 studies), Alpha-2-macroglobulin (A2MG, 4 studies), FIBB (3 studies), Pyruvate kinase (KPYM, 3 studies) and Spondin-1 (SPON1, 3 studies). This set of proteins has previously been reported to increase in AD CSF [13]. However, our analysis, which includes only significant data, permits the identification of proteins with consistently increased levels in AD CSF whose potential as reliable AD biomarkers was previously overlooked. For example, Clusterin (CLUS) was found to be increased in AD in 6 out of 7 studies. This chaperone protein is involved in lipid transport and metabolism and is produced and secreted predominantly by astrocytes within the CNS [28]. Various studies of CSF from AD patients, most using an ELISA approach, have proposed a role for CLUS as a potential AD biomarker [reviewed in [24]]; our study now provides additional support for this idea.

Finally, SPARC, also known as Osteonectin, was found to be consistently upregulated in CSF from AD patients (3 studies), which validates its novel utility as a reliable CSF biomarker. In brain, its expression is restricted to microglia and subcortical astrocytes, and a role for SPRC has been suggested in neuroinflammation [29, 30]. Specifically, high levels of SPRC have been shown in AD brain wherein it colocalizes to Aβ protein depots. It has been proposed that SPRC contributes to cerebral inflammation and subsequent tissue repair [31].

### Proteins consistently reduced in AD CSF

Our analysis of proteins consistently reduced in AD identified 19 proteins representing the most consistent biomarkers (bolditalic proteins in Table 4). With our experimental approach, we identified 3 proteins previously reported by Wesenhagen et al., Neurexin 1A (NRX1A), APOC2 and FBLN3. In our study, NRX1A [and Neurexin-2 (NRX2A) and Neurexin-3-beta (NRX3B) to a lesser degree] appears consistently reduced in AD CSF across all of the 4 independent studies in which it was identified. These brain-specific proteins participate in synapse formation, plasticity and stability [32]. A recently published targeted proteomic study using Selected Reaction Monitoring, which monitors neurexin levels in control and preclinical AD patients, reinforces the data reported here by showing reduced levels of NRX2A and NRX3B in preclinical AD CSF [33]. Overall, the reduced levels of neurexins observed in CSF at the earliest preclinical AD stages support the idea of diminished synaptic density during AD progression. We also observed a downregulation of APOC2 in CSF of AD patients in 3 independent studies, again strengthening the notion that a change in lipid metabolism can be potentially related to cognitive status in AD. Supporting this idea, our study found Apolipoprotein A1 *(*APOA1) as consistently reduced in AD CSF (7 out of 8 studies). Considering that APOA1 is among the most abundant proteins within human CSF [18], the use of APOA1 as biomarker of AD progression is especially relevant to practical biomarker identification, as its reduction in the context of AD is easily measurable.

Finally, FBLN3 codified by the gene *EFEMP1*, is a glycoprotein associated with the extracellular matrix which is involved in cell proliferation and migration [34]. Although no direct evidence as to a specific role for FBLN3 in AD has been reported, FBLN3 has recently been described as an amyloidogenic protein [35]. FBLN3 was observed to be consistently downregulated in CSF from AD patients (3 studies). Interestingly, only one tryptic peptide (QTSPVSAMLVLVK) was observed to be down-regulated in all 3 studies, thus highlighting this peptide as the most relevant hit for the characterization of AD status.

Together with VGF, the members of the granin family Chromogranin-A (CMGA), Secretogranin-1 (SCG1), SCG2, Secretogranin-3 (SCG3) and PCSK1N were found to be consistently decreased in our study. CMGA has been the most frequently detected granin in proteomic CSF studies (7 studies), and this protein, together with SCG1 (found to be decreased in 4 out of 5 studies) represent the most abundant granins in human CSF. Immunoreactivity for CMGA, SCG1 and SCG2 has been observed in amyloid plaques of post-mortem brains from AD patients [36–39]. Taken together, the proteomic studies gathered in this review support the role of CMGA as a reliable CSF biomarker. It is especially relevant that the most consistent tryptic peptides of all proteins that decrease in AD correspond to granins (VGF, CMGA, SCG1, SCG2 SCG3, and PCSK1N). Their acidic isoelectric point and the presence of multiple dibasic cleavage sites could potentially favor their detection by MS-spectrometry, which might constitute an assay advantage, since a panel of these peptides could be designed as a direct tool for a fast spectrometric characterization of AD patients.

The granin family member PCSK1N, known as proSAAS, is a protein produced almost exclusively by neurons and endocrine cells, and was reduced in 6 out of the 8 AD proteomic studies where it was identified. Reduced PCSK1N levels in CSF may be related to increased brain retention of this protein within plaques and other aggregates, as previously observed [40–42]. In agreement with this idea, a recent transcriptomic study [43] found increased PCSK1N expression during AD progression. Interestingly, among all of the peptides included in our peptidomic study, the proSAAS peptide ETPAPQVPARRLLPP was the most consistent finding and corresponds to the C-terminal sequence known as BigLEN (LETPAPQVPARRLLPP). There is a consistent lack of the N-terminal Leucine on the retrieved peptides [15, 44–46] which suggests that this peptide corresponds to a biological fragment of proSAAS in CSF not resulting from tryptic cleavage, emphasizing the importance of this modified BigPEN peptide as a possible direct biomarker of AD in CSF.

Interestingly, both Neuronal pentraxin-1 (NPTX1) and Neuronal pentraxin receptor (NPTXR) showed consistent reduction (4 and 4 out of 5 studies, respectively). These proteins have been previously implicated in AD [47]. Although NPTX1 has been primarily considered as a plasma biomarker [48], the current analysis supports its role as a reliable CSF AD biomarker. As this protein likely constitutes a protein associated with both neuronal degradation and synaptic loss [49, 50], future work will be needed to determine the specificity of NPTX1 for AD versus other neurodegenerative diseases. The potential role of the NPTXR as a prognostic biomarker for AD has recently been studied by others [20, 51]. Begcevic et al., observed that CSF NPTXR levels decrease with the severity of AD [51], thereby supporting the significance of NPTXR as a valuable CSF biomarker for specific stages of AD progression. It is important to point out that from all proteomic studies included in our analysis, NPTX1 and NPTXR were identified together in 2 studies [52] which reinforces the importance of data-mining efforts to identify and consolidate reliable biomarkers of neurodegeneration.

MUC18, an adhesion molecule encoded by the *MCAM*-*CD14* gene, was consistently reduced in AD CSF in 4 independent proteomic studies. We believe this hit is especially interesting since this protein has not been previously linked to the disease. MUC18 is expressed by a subpopulation of IL-17-secreting CD4 + and CD8 + human T cells (Th17 and Tc17 cells, respectively) [53–55]. Chronic neuroinflammation is a phenomenon commonly observed in AD [56], and various Th cell lineages, including Th1, Th17 and regulatory T cells, appear to play a complex role in AD-associated neurodegeneration [57–62]. In order to explain the significance of reduced MUC18 levels in AD CSF, we suggest that further study of the role of MUC18 in AD inflammation is important.

The Retinol-binding protein 4 (RBP4), found to be reduced in 4 out of 5 studies, can circulate as an adipokine, and is related to insulin metabolism and retinoic acid signalling, both AD-associated processes [63, 64]. Additionally, RBP4 binds to TTHY, a protein that is believed to modulate Aβ levels by transporting Aβ from brain to the periphery [65]. Studies in patients have shown reduced levels of RBP4 in AD [66–68]. Indeed, this decrease is associated with cognitive decline, suggesting that RBP4 might be a biomarker for AD progression [66–68]. Nevertheless, it has been recently shown that RBP4 levels are not altered in preclinical AD CSF samples [63], implying that RBP4 may not be a good biomarker at preclinical stages.

The Amyloid-like protein 1 (APLP1), a member of the APP family, was found to consistently decrease in 3 independent studies. This neuron-specific protein [69] is involved in the maintenance of dendritic spines and basal synaptic transmission [70]. APLP1 is a γ-secretase substrate [71], thereby, secreted APLP1 fragments might be of especial interest to investigate γ-secretase cleavage products in AD.

The actin-binding protein Gelsolin (GELS), which was consistently decreased in AD in 3 independent studies, has already been implicated in AD [72]. GELS specifically binds to Aβ, inhibits its aggregation and protects cells from Aβ-induced apoptosis [72]. Indeed, its role as a potential therapeutic strategy for AD treatment is currently being evaluated [73, 74].

The essential component of the coagulation pathway KNG1 [75], was consistently reduced in AD CSF in 3 independent proteomic studies. KNG1 is particularly attractive for further investigation since a direct relation between KNG1 and AD has not yet been reported. However, the only study which investigated KNG1 in AD (since KNG1 polymorphisms were shown to be associated with hypertension, and thereby, they hypothesized that could be cause for AD progression) showed that KNG1 polymorphisms are not correlated with the incidence of late onset AD in a 201 patient cohort [76].

Metallothionein-3 (MT3) was found to be consistently reduced in AD CSF (3 studies). This protein regulates CNS Cu^2+^ and Zn^2+^ transport and storage and inhibits the toxicity of these metals, thus representing a major component of metal homeostasis [77]; this protein thus may play an important role in AD progression.

Finally, Superoxide dismutase (SODC), which neutralizes superoxide oxygen radicals to hydrogen peroxide and molecular oxygen inside the cells [78], appeared to be consistently downregulated in CSF from AD patients (3 studies). Several studies have highlighted the role of SODC deficiency in the acceleration of Aβ oligomerization, neuronal inflammation, and memory impairment in AD [79, 80], thus establishing SODC as an important marker in the etiopathogenesis of this disease.

Given that we only included significant data in our study, the finding that the transcription factor NFE2L2/NRF2 was identified as the most important upstream regulator predicted to be repressed in AD patients is especially interesting. A reduction in this transcription factor could potentially result in several of the observed proteomics changes in AD [specifically underlying the reduced levels of Angiotensinogen (AGT), Complement factor D (CFAD), SCG1, Collagen alpha-1(I) chain (CO1A1), Peptidyl-prolyl cis–trans isomerase B (PPIB), SCG2, SODC and Extracellular superoxide dismutase (SODE) expression]. In this regard, it has been recently shown that NRF2 deficiency replicates the transcriptomic changes seen in Alzheimer’s patients and worsens APP and tau pathology [81]. Interestingly, we also identified STK11 [also known as liver kinase B1 (LKB1)], as an upstream regulator predicted to be activated in AD patients. STK11 has been described as a multifunctional master kinase which is involved in a variety of functions in the nervous system such as maintaining axon integrity, neural development, neural homeostasis, neuronal survival, and control of neurotransmitter release [82]. Indeed, its deletion leads to axon degeneration [83]. Additionally, dysregulation of STK11 has been shown to contribute to Aβ accumulation and tauopathy AD-associated [84, 85].

## Conclusions

Data-mining of CSF proteomic studies from individuals suffering from AD retrieves and consolidates valuable information as to proteins and peptides clearly altered in AD, information that may be useful in the constitution of a screening panel to increase the accuracy of AD diagnosis. From a methodological perspective, there are still several challenges to solve, as it is unclear which proteins or specific peptides can readily be measured in patient samples; although ELISA would work for several candidates, the low abundance of other candidates (detected exclusively by mass spectrometry) renders their quantification directly in CSF more complex. Nevertheless, the evaluation of information regarding specific peptides has not been previously performed, and the data we present herein provides direct targets towards establishing the status of a given protein in AD. These data should support the development of a peptide array with verified biomarker candidates that could move into clinical practice, even within the next few years, to fulfill the need for early detection in order to better combat this widespread neurodegenerative disease. Names of the genes and proteins cited in the text are described in Tables 3, 4 and 5.

## Supplementary information

**Additional file 1: Table S1.** Information regarding all the proteins extracted from the proteomic studies. The proteins identified in each article and their abbreviations are shown. Δ, proteins not recognized by DAVID bioinformatics resources, Π qualitative but no quantitative data.

**Additional file 2: Table S2.** Proteins identified with significant changes between AD and control samples. The number of studies that describe these changes are shown.

**Additional file 3: Table S3.** Proteins showing inconsistent changes in AD samples. $ proteins which are abundant in the CSF of healthy individuals [18]. Component CO4A and CO4B are indicated as A and B respectively.

**Additional file 4: Table S4.** Information regarding all peptides extracted from the proteomic studies. M.W. [Da]: Molecular weight obtained from each article. Theo. M.W. [Da]: theoretical molecular weight obtained from Compute pI/Mw tool (https://web.expasy.org/compute_pi/).

**Additional file 5: Table S5.** Peptide sequences showing inconsistent changes in CSF studies. Peptide sequences with different pattern of expression across independent studies or inconsistent direction change to that observed by proteomics in relation to AD pathology. *Peptide sequences with changes supported by statistical analysis between AD and control samples.

**Additional file 6: Figure S1.** Most frequently detected peptides within 13 protein sequences. Peptide sequences with the same pattern of expression in at least 2 (bold red) or 3 (bold red underlined) independent studies and maintain consistent direction change to that observed by proteomics in relation to the AD pathology.

## Data Availability

Not applicable.

## Abbreviations

AD: Alzheimer´s disease
ADP: Adenosine diphosphate
AR: Age range
Aβ42: Amyloid-beta 1-42
BBB: Blood-brain barrier
CDR: Clinical dementia rating scale
CE-MS: Capillary electrophoresis mass spectrometry
CERAD: Consortium to Establish a Registry for Alzheimer’s Disease
CNS: Central nervous system
CSF: Cerebrospinal fluid
CT: Computed tomography
DAVID: Database for annotation, visualization and integrated discovery
DA: Daltons
DNA: Deoxyribonucleic acid
DSM: Diagnostic and statistical
ELISA: Enzyme-linked immunosorbent assay
ESI: Electrospray ionization
FAD: Familial alzheimer Disease
IAPP: Islet amyloid polypeptide
ICD-10: 10th revision of the International Statistical Classification of Diseases and Related Health Problems
ID: Identifier digital
IWG-2: International Working Group-2 criteria
IPA: Ingenuity pathway analysis
LC–MS/MS: Liquid chromatography-tandem mass spectrometry
MAP-RBM: Multi-analyte profiling technology platform- rules based medicine
MCI: Mild cognitive impairment
MCs: Mutation carriers
MEDLINE: Medical literature analysis and retrieval system online
MMSE: Mini-menta state exam
MOCA: Montreal cognitive assessment
MRI: Magnetic resonance imaging
MRM: Multiple reaction monitoring
mRNA: Messenger ribonucleic acid
MS: Mass spectrometry
NA: Not Applicable
NCs: Mutation non-carriers
NI: Not information
NIA-AA: National Institute on Aging and Alzheimer’s Association
NINCDS-ADRDA: National Institute of Neurological and Communicative Disorders and Stroke and the AD and Related Disorders Association
PRM: Parallel reaction monitoring
PSEN1: Presenilin 1
p-tau: Phosphorylated tau
RNA: Ribonucleic acid
ROS: Reactive oxygen species
RP-LC MS/MS: Reversed-phase LC–MS/MS
SELDI-TOF-MS: Surface enhanced laser desorption/ionization time-of-flight mass spectrometry
SMC: Subjective memory complaints
TMT: Tandem mass tag
T-tau: Total tau
WB: Western blot
