# Supplementary material for: A comprehensive systematic review of CSF proteins and peptides that define Alzheimer’s disease
Source: Clin Proteomics. 2020 Jun 5;17:21. doi: 10.1186/s12014-020-09276-9 (PMC7273668; doi:10.1186/s12014-020-09276-9)
Supplement: Supplementary file 6 — Additional file 6: Figure S1. Most frequently detected peptides within 13 protein sequences. Peptide sequences with the same pattern of expression in at least 2 (bold red) or 3 (bold red underlined) independent studies and maintain consistent direction change to that observed by proteomics in relation to the AD pathology. [file 12014_2020_9276_MOESM6_ESM.pdf]

MS-Peptides consistently increased (proteins from data Analysis 2)

CH3L1

MGVKASQTGFVVLVLLQCCSAYKLVCYYTSWSQYREGDGSCFPDALDRFLCTHIIYSFANISNDHIDTWEWNDVTLYGMLNTLKNRNPNLK**TLLSVGGWNFGSQR**FSKIASNTQSRRTFIKSVPPFLRTHGFDGLD
LAWLYPGRGDKQHFTTLIKEMKAEFIKEAQPGKKQLLLSAALSAGKVTIDSSYDIAKISQHLDFISIMTYDFHGAWRGTTGHHSPLFRGQEDASPDRFSNTDYAVGYMLRLGAPASK**LVMGIPTFGR**SFTLASSET
GVGAPISGPGIPGRFTK**EAGTLAYYEICDFLR**GATVHRILGQQVPYATKGNQWVGYDDQESVKSKVQYLKDRQLAGAMVWALDLDDFQGSFCGQDLRFPLTNAIKDALAAT

CLUS

MMKTLLLFVGLLLTWESGQVLG**DQTVSDNELQEMSNQGSKYVNKE**IQNAVNGVKQIKTLIEKTNEERKTLLSNLEEAKKKKEDALNETRESETKLKELPGVCNETMMALWEECKPCLKQTCMKFYARVCRSGSGLV
GRQLEEFLNQSSPFYFWMNGDRIDSLLENDRQQTHMLDVMQDHFSRASSIIDELFQDRFFTREPQDTYHYLPFSLPHRRPHFFFPKSRIVRSLMPFSPYEPLNFHAMFQPFLEMIHEAQQAMDIHFHSPAFQHPPT
EFIREGDDDRTVCREIRHNSTGCLRMKDQCDKCREILSVDCSTNNPSQAKLRRELDESLQVAERLTRKYNELLKSYQWKMLNTSSLLEQLNEQFNWVSRLANLTQGEDQYYLRVTTVA**SHTSDSDVPSGVTEV**VVK
L**FDSDPITVTVPVEVSRKNPK**FMETVAEKALQEYRKKHREE

MS-Peptides consistently decreased (proteins from data Analysis 2)

VGF

MKALRLSASALFCLLLINGLGA**APPGRPEAQPPPLSSEHKEPVAGDAVPGPKDGSAPEVRGA**R**NSEPQDEGELFQGVDPRALAAVLLQALDRPASPPAPSGSQQGPEEEAAEALLTETV**RSQTHSLPAPESPEPAAP
PRPQTPENGPEASDPSEELEALASLLQELRDFSPSSAKRQQETAAAETETRTHTLTRVNLESPGPERVWRASWGEFQARVPERAPLPPPAPSQFQARMPDSGPLPETHKFGEGVSSPKTHLGEALAPLSK**AYQGVAA**
PFPKARRPESALLGGSEAGERLLQQGLAQVEAGRRQAEATR**QAAAQEER**LADLASDLLLQYLLQGGARQRGLGGR**GLQEAAEER**ESAREEEEAEQERR**GGEERVGEEDEEAAEAEAEAEEAERARQNA**LLFAEEEDG
EAGAEDKRSQEETPGHRRKEAEGTEEGGEEEDDEEMDPQTIDSLIELSTKLHLPADDVVSIIEEVEEKRKRK**KNAPPEPVPPPRAAPAPTHVRSP**QPPPPAPAPARDELPDWNEVLPPWDREEDEVYPPGPYHPFPN
YIRPRTLQPPSALRRRHYHHALPPSRHYPGREAQARR**AQEEAEAEERRLQEQEELEN**YIEHVLLRRP

SCG2

MAEAKTHWLGAALSLIPLIFLISGAEAASFQRNQLLQKEPDLRLENVQK**FPSPEMIRALEYIENLR**QQAHKEESSPDYNPYQGVSVPLQQKENGDESHLPER**DSLSEEDWMR**IILEALR**QAENEPQSAPK**ENKPYAL
NSEKNFPMDMSDDYETQQWPERKLKHMQFPPMYEENSRDNPFKR**TNEIVEEQYTPQSLATLESVFQELGKLTGPNNQ**KRERMDEEQKLYTDDEDDIYKANNIAYEDVVGGEDWNPVEEK**IESQTQEEVRDSK**ENIGK
NEQINDEMKRSGQLGIQEEDLRKESKDQLSDDVSKVIAYLK**RLVNAAGSGR**LQNGQNGERATRLFEKPLDSQSIYQLIEISRNLQIPPEDLIEMLKTGEKPNGSVEPERELDLPVDLDDISEADLDHPDLFQNRMLS
KSGYPKTPGRAGTEALPDGLSVEDILNLLGMESAANQKTSYFPNPYNQEKVLPRLPYGAGRSRSNQLPKAAWIPHVENRQMAYENLNDKDQELGEYLARMLVKYPEIINSNQVKR**VPGQGSSEDDLQEEEQIEQAIK**
EHLNQGSSQETDKLAPVSKRFPV**GPPKNDDTPNRQ**YWDEDLLMK**VLEYLNQEK**AEKGREHIAKRAMENM

SCG3

^{35}**ELSAERPLNEQIAEAEEDKI**^{54}

CMGA

MRSAAVLALLLCAGQVTALPVNSPMNKGDTEVMKCIVEVISDTLSKPSPMPVSQECFETLRGDERILSILRHQNLLKELQDLALQGAKERAHQQKKHS**GFEDELSEVLENQSSQAELKEAVEEPSSKDVME**KREDSK
EAEKSGEATDGARPQALPEPMQESKAEGNNQAPGEEEEEEEEATNTHPPASLPSQK**YPGPQAEGDSEGLSQGLVDR**EKGLSAEPGWQAKREEEEEEEEEAEAGEEAVPEEEGPTVVLNPHPSLGYKEIRKGESRSEA
LAVDGAGKPGAEEAQDPEGKGEQEHSQQKEEEEEMAVVPQGLFRGGKSGELEQEEERLSKEWEDSKRWSKMDQLAKELTAEKR**LEGQEEEEDNRDSSMKLSFRA**RAYGFRGPGPQLRRGWRPSSREDSLEAGLPLQV
RGYPEEKKEEEGSANRRPEDQELESLSAIEAELEKVAHQLQALRRG

NPTXR

^{278} **VAELEHGSSAYSPPDAFK** ^{295}

PCSK1N

MAGSPLLWGPRAGGVGLLVLLLLGLFRPPPALCARPVKEPRGLSAA**SPPLAETG**APRRFRRSVPRGEAAGAVQELARALAHLLEAERQERARAEAQEAEDQQARVLAQLLRVWGAPRNSDPALGLDDDPDAPAAQL
ARALLRARLDPAALAAQLVPAPVPAAALRPRPPVYDDGPAGPDAEEAGDETPDVDPELLRYLLGRILAGSADSEGVAAPRRLRRAA**DHDVGSELPPEGVLGA**LLRVKRL**ETPAPQVPARRLLPP**

APLP1

MGPASPATRGLGRRRGPPPLPLLLPLSLLLLRAQLAVG**SLAGGSPGAAEAPGSAQVAGLCGRLT**LHRDLRTGRWEPDPQRSRRCLRDPQRVLEYCRQMYPELQIARVEQATQAIPMERWCGGARGGRCAHPHHQVV
PFHCLPGEFVSEALLVPEGCRFLHQERMDQCESSTRRHQEAQEACSSQGLILHGSGMLLPCGTDRFRGVEYVCCPPPVTPNPSGTAVGDPSTRSWPPGSRVEGVEDEEEEESFLQPVDDYFVEPPRAEEEEEEKAP
PSSSHTPAGVSKVCSRKCSSQGRKSDLEMSFSLTPIPGLAVTPTPRPTDGVDVYFGMPGEISEHEGFLRAKMDLEERRMRQINEVMREWAMADNQSKNLPKADRQALNEHFQSILQTLEEQVSGERQRLVETHATR
VIALINDQRRAALEGFLAALQGDLPQPERVLLALRRYLRAEQKEQRHTLRHYQHVAAVDPEKAQQMRFQVQTHLQVIEERMNQSLGLLDQNPQLAQELRPQIQELLHSEHLGPNELEAPAPAGSSEDKGELQPLDS
KDADTPMALPKGSTEQDAASPGKENMSPLEQYERKVNVSVPR**GFPFHSSEIQ**R**DELAPAGTGVSREAVSGLLIMGAGGGSL**IVLSLLLLRRKKPYGAISHGVVEVDPMLTLEEQQLRELQRHGYENPTYRFLEERP

APOC2

MGTRLLPALFLVLLVLGFEVQGTQQPQQDEMPSPTFLTQVK**ESLSSYWESAKTAAQNLYEKTYLPAVDEK**LRDLYSKSTAAMSTYTGIFTDQVLSVLKGEE

FBLN3

MLKALFLTMLTLALVKSQDTEETITYTQCTDGYEWDPVRQQCKDIDECDIVPDACKGGMKCVNHYGGYLCLPKTAQIIVNNEQPQQETQPAEGTSGATTGVVAASSMATSGVLPGGGFVASAAAVAGPEMQTGRNNFV
IRRNPADPQRIPSNPSHRIQCAAGYEQSEHNVCQDIDECTAGTHNCR**ADQVCINLR**GSFACQCPPGYQKRGEQCVDIDECTIPPYCHQRCVNTPGSFYCQCSPGFQLAANNYTCVDINECDASNQCAQQCYNILGSFI
CQCNQGYELSSDR**LNCEDIDECR**TSSYLCQYQCVNEPGKFSCMCPQGYQVVRSR**TCQDINECETTNECR**EDEMCWNYHGGFRCYPR**NPCQDPYILTPENR**CVCPVSNAMCRELPQSIVYKYMSIRSDRSVPSDIFQIQ
ATTIYANTINTFRIK**SGNENGEFYLRQTSPVSAMLVLVK**SLSGPREHIVDLEMLTVSSIGTFRTSSVLRLTIIVGPFSF

GELS

^{584}**AGALNSNDAFVLK**TPSAAYLWVGTGASEAEK**TGAQELLR**VLRAQPVQVAEGSEPDGFWEALGGKAAYRTSPRLKDKKMDAHPPRLFACSNKIGRFVIEEVPGELMQEDLATDDVMLLDTWDQVFVWVGKDSQEEEK
TEALTSAKRYIETDPANRDRRTPITVVKQGFEPPSFVGWFLGWDDDYWSVDPLDRAMAELAA

SCG1

^{292}**SSQGGSLPSEEKGHPQEESEESN**VSMASLGEKRDHHSTHYRASEEEPEYGEEIKGYPGVQAPEDLEWERYRGRGSEEYRAPRPQSEESWDEEDKRNYPSLELDKMAHGYGEESEEERGLEPGKGRHHRGRGGEPRA
YFMSDTREEKRFLGEGHHRVQENQMDKARRHPQGAWKELDRNYLNYGEEGAPGKWQQQGDLQDTKENREEARFQDKQYSSHHTAEKRKRLGELFNPYYDPLQWKSSHFERRDNMNDNFLEGEEENELTLNEKNFFPEY
NYDWWEKKPFSEDVNWGYEKRNLARVPKLDLKRQYDRVAQLDQLLHYRKKSAEFPDFYDSEEPVSTHQEAENEKDR**ADQTVLTEDEKKELENLAAMDLELQK**IAEKFSQRG
